# Soft X-rays with orbital angular momentum for resonant scattering experiments at Synchrotron SOLEIL

**DOI:** 10.1107/S160057752600322X

**Published:** 2026-04-20

**Authors:** Pietro Carrara, Franck Fortuna, Renaud Delaunay, Joan Vila-Comamala, Benedikt Rösner, Christian David, Stefania Pizzini, Clément Fourniols, Laurent Vila, Matteo Pancaldi, Carlo Spezzani, Flavio Capotondi, Pierre Nonnon, Mauro Fanciulli, Thierry Ruchon, Nicolas Jaouen, Horia Popescu, Maurizio Sacchi

**Affiliations:** a Sorbonne Université CNRS, Institut des NanoSciences de Paris, INSP 75005Paris France; b Université Paris-Saclay CNRS, Institut des Sciences Moléculaires d’Orsay 91405Orsay France; c Sorbonne Université CNRS, Laboratoire de Chimie Physique—Matière et Rayonnement, LCPMR 75005Paris France; d Center for Photon Science Paul Scherrer Institute 5232Villigen PSI Switzerland; e Université Grenoble Alpes CNRS, Institut Néel 38042Grenoble France; f Université Grenoble Alpes CNRS, CEA, Grenoble INP, IRIG-SPINTEC 38000Grenoble France; g Elettra-Sincrotrone Trieste Strada Statale 14 km 163,5 in Area Science Park Basovizza 34012Trieste Italy; h CY Cergy Paris Université CEA, LIDYL 91191Gif-sur-Yvette France; i Université Paris-Saclay CEA, LIDYL 91191Gif-sur-Yvette France; j Synchrotron SOLEIL Saint-Aubin, Boite Postale 48 91192Gif-sur-Yvette France; POSTECH, Republic of Korea

**Keywords:** soft X-rays with orbital angular momentum, resonant scattering, Synchrotron SOLEIL

## Abstract

The paper presents a comprehensive description of a new setup implemented and commissioned at the SEXTANTS beamline of Synchrotron SOLEIL for absorption and scattering experiments with X-ray beams carrying an orbital angular momentum, also known as *twisted*X-ray beams. Two alternative methods have been implemented, based on the use of either spiral zone plates or fork grating devices, and we show how they can be used for both defining and assessing the orbital angular momentum of an X-ray beam. We also demonstrate that cascading multiple devices enables integer operations on the orbital angular momentum of the resulting X-ray beam. Finally, we report the results of the first resonant scattering pilot experiments in transmission and reflection mode, intended to assess the feasibility of future users’ measurements. The availability of twisted soft X-rays complements the range of experimental techniques in elastic, resonant and coherent scattering available at the SEXTANTS beamline.

## Abstract 2

Characterization and testing of a new setup for absorption and scattering experiments with soft X-rays carrying an orbital angular momentum at the SEXTANTS beamline of Synchrotron SOLEIL are presented.

## 1. Introduction

In addition to the spin angular momentum that originates from light polarization, photon beams can also carry an orbital angular momentum (OAM) of **L**ℏ per photon, with **L** ∈ 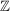, associated with an azimuthally varying phase term exp(*i***L**ϕ) in the Laguerre–Gaussian representation of the electromagnetic radiation (Allen *et al.*, 1992 ▸). In the visible and near-infrared (vis-IR) range of wavelengths, OAM laser beams, also called *twisted optical beams*, find applications in fields as varied as biology, telecommunications, imaging and quantum technologies (Shen *et al.*, 2019 ▸). Their capability to exert a mechanical torque has been exploited to create *optical spanners* for manipulating small particles (Simpson *et al.*, 1997 ▸; Ladavac & Grier, 2004 ▸). The azimuthal phase dependence introduces a singularity on the propagation axis and a radial modulation of the intensity, which results in a ring-shaped beam. Such properties have been used to modify magnetic ordering (Fujita & Sato, 2017 ▸), to improve the spatial resolution in microscopy (Tamburini *et al.*, 2006 ▸), and to enhance the edge sharpness in phase contrast imaging (Fürhapter *et al.*, 2005 ▸). More recently (Géneaux *et al.*, 2016 ▸; Gauthier *et al.*, 2017 ▸; Rebernik Ribič *et al.*, 2017 ▸), the generation of OAM beams at shorter wavelengths, from extreme ultraviolet (XUV) to hard X-rays, is also finding an increasing number of applications, often based on extrapolations of previous work carried out in the vis-IR range. For instance, a recent study by Pancaldi *et al.* (2024 ▸) compared XUV ptychographic images of a reference sample obtained using beams with different OAM values, confirming that the attainable spatial resolution improves with **L**. Extending the use of OAM beams from the vis-IR (Tamburini *et al.*, 2006 ▸) to the XUV (Wang *et al.*, 2023 ▸; Pancaldi *et al.*, 2024 ▸) and X-rays opens new perspectives for high-resolution element-selective X-ray imaging.

X-ray radiation carrying orbital angular momentum (X-OAM) has received increasing interest over the last few years, both in terms of theoretical studies (Nazirkar *et al.*, 2024 ▸; Yan & Geloni, 2023 ▸; Moghaddasi Fereidani *et al.*, 2025 ▸) and of practical applications in different fields of research (Fujita & Sato, 2017 ▸; Loetgering *et al.*, 2020 ▸; Woods *et al.*, 2021 ▸; McCarter *et al.*, 2023 ▸). As with the spin angular momentum, the handedness imposed by the OAM has been exploited to perform spectroscopic studies of chiral molecules with hard X-rays (Rouxel *et al.*, 2022 ▸) and of magnetic materials in the XUV range (Fanciulli *et al.*, 2022 ▸; Fanciulli *et al.*, 2025 ▸). In particular, the concept of magnetic helicoidal dichroism has been introduced to describe the dependence of the resonant absorption and scattering from a magnetic material on the OAM value of the incident photon beam (Fanciulli *et al.*, 2021 ▸; Ruchon *et al.*, 2022 ▸; Luttmann *et al.*, 2025 ▸).

Studies with X-OAM beams are recent and still relatively scarce, but interest in the field is clearly growing (Ye *et al.*, 2019 ▸; Rouxel *et al.*, 2022 ▸; Jiang *et al.*, 2023 ▸; Venkatesh *et al.*, 2026 ▸). Here we present a new setup dedicated to absorption and scattering experiments with soft X-rays carrying OAM, which has been commissioned at the SEXTANTS beamline (Sacchi *et al.*, 2013*a* ▸) of the Synchrotron SOLEIL source, to complement the range of techniques in elastic, resonant and coherent scattering available to the users. We implemented two ways of producing X-OAM beams, using either spiral zone plates (SZPs; Vila-Comamala *et al.*, 2014 ▸) or fork gratings (FGs; Lee *et al.*, 2019 ▸), and explored the possibility of generating X-OAM radiation directly from helical undulator sources (Sasaki *et al.*, 2007 ▸; Sasaki & McNulty, 2008 ▸). Finally, we present the results of flux-demanding test measurements over the 130–1200 eV range, performed using these X-OAM beams.

## 2. Description of the experimental setup

The IRMA-2 endstation (Sacchi *et al.*, 2013*b* ▸) was installed at the end of the SEXTANTS elastic scattering branch (Sacchi *et al.*, 2013*a* ▸). Approximately 9 m upstream of the endstation, the beamline features an intermediate focal point where a pin-hole (10, 20 or 50 µm in diameter) can be inserted to create a secondary source, trimming the beam and ensuring a high degree of transverse coherence. Downstream of the secondary source, a Kirkpatrick–Baez (KB) refocusing optics with bendable mirrors, placed approximately 3 m before the IRMA-2 chamber, allows for either a large (≥2 mm) or focused (∼40 µm × 30 µm, FWHM horizontal × vertical) beam to illuminate the SZP or the FG, respectively.

**Figure 1:**
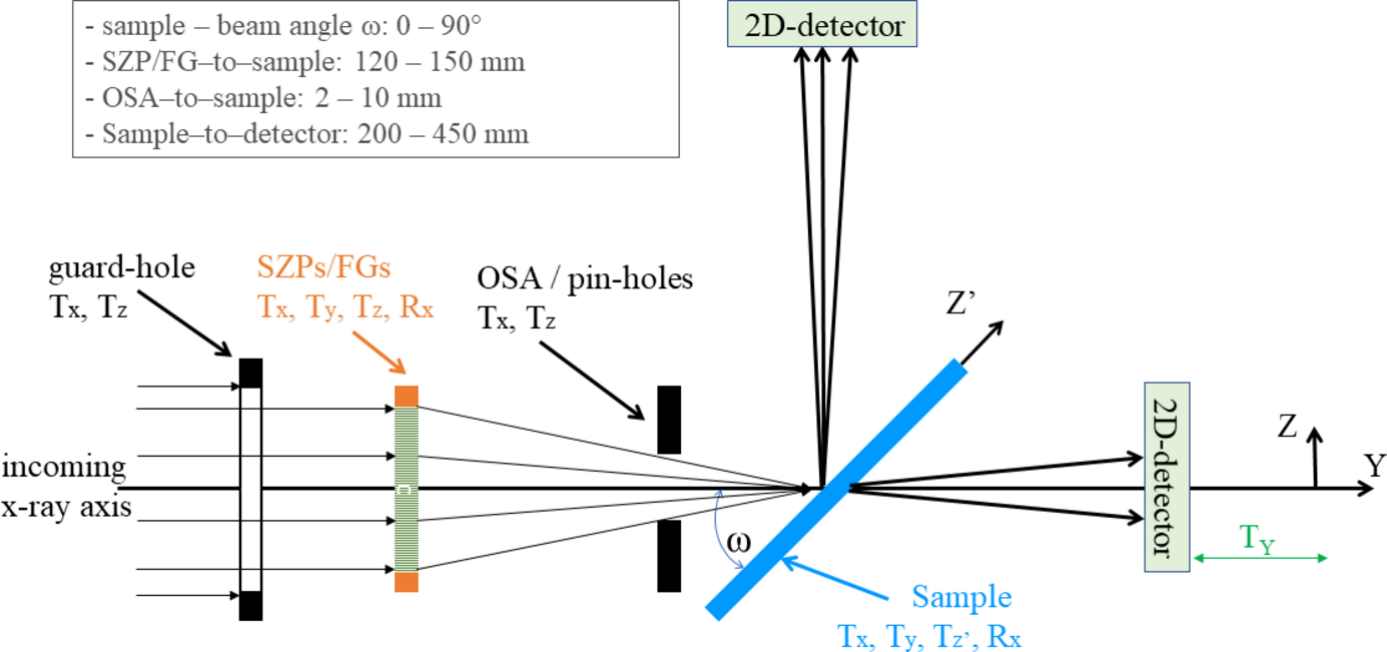
Side-view sketch (not to scale) of the experimental setup inside the IRMA-2 chamber.

The experimental setup, sketched in Fig. 1 ▸, consists of the following elements:

(i) a fixed 2.5 mm circular aperture for skimming the beam before the experimental chamber; this is especially important under defocused KB conditions for SZP illumination;

(ii) encoded XYZ-tables driven by piezoelectric motors (100 nm accuracy in all three directions), with additional *R*_*X*_ manual alignment, hosting the SZPs and/or the FGs;

(iii) encoded piezo-driven XYZ-tables (100 nm accuracy) for aligning a set of order-sorting apertures (OSAs) from 20 µm to 1 mm in size;

(iv) high-precision XYZR_X_ tables (accuracy, after backlash correction, of 100 nm for the translations and 0.01° for the rotation, with translation ranges ≥ 25 mm) driven by stepper motors for aligning samples (or a second set of FGs);

(v) A long-travel translator fixed on a CF-150 flange carrying an in-vacuum CCD camera (PI-MTE with 2048 × 2048 pixels, 13.5 µm × 13.5 µm in size) equipped with a light-tight Zr filter. The sample-to-CCD distance can be varied between 200 and 450 mm, allowing for a trade-off between the angular range covered by the detector and the angular resolution of a single pixel, depending on the specific needs of each experiment. A beam-stop can be positioned over the detector active area using two piezo-driven translation tables attached to the CCD frame.

The detection assembly can be mounted on the IRMA-2 chamber either in a horizontal or in a vertical position to measure in transmission or reflection geometry, respectively. Positioning the CCD at intermediate angles (ranges 30 ± 10° and 60 ± 10°) is also possible using CF-100 mounting flanges (Sacchi *et al.*, 2013*b* ▸), but keeping a fixed distance of 450 mm from the sample. For alignment purposes, the radiation intensity, either transmitted or reflected, can also be measured by retractable photodiodes placed in between the sample and the CCD.

With the exception of results shown in Appendix *A*[App appa], the test measurements reported here were performed in transmission mode.

## 3. Spiral zone plates

SZPs are diffractive focusing elements that, in addition to producing a highly demagnified and convergent beam, also impart OAM to the X-rays. The **L** value of the outgoing beam is determined by the number ***s*** of spiraling branches (Vila-Comamala *et al.*, 2014 ▸), with the sign of ***s*** defining whether the spirals turn clockwise (CW) or counter-clockwise (CCW) with respect to the wavevector of the incoming photons. SZP devices have been tested with X-rays for a few years already in experiments performed at synchrotrons (Cojoc *et al.*, 2006 ▸; Kohmura *et al.*, 2020 ▸; Rouxel *et al.*, 2022 ▸) and free-electron lasers (FELs) (Fanciulli *et al.*, 2022 ▸; Pancaldi *et al.*, 2024 ▸).

**Figure 2:**
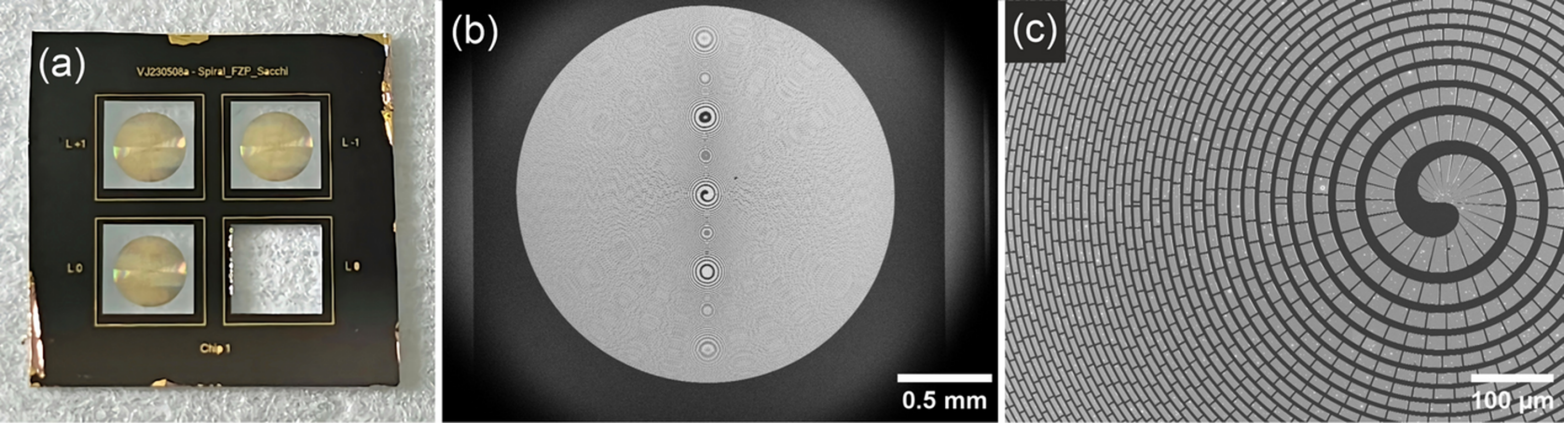
(*a*) Photograph of a 10 mm Si frame hosting three SZPs with ***s*** = −1, 0 and +1. (*b*, *c*) Scanning electron microscopy (SEM) images of the ***s*** = +1 SZP with different magnifications [scalebars are 0.5 mm in (*b*) and 0.1 mm in (*c*)].

SZPs with ***s*** = 0, ±1, ±2, ±3 were manufactured at the Laboratory for X-ray Nanoscience and Technologies of the Paul Scherrer Institut (Villigen, Switzerland) by electron-beam lithography, Au thermal evaporation, and lift-off process on silicon nitride (Si_3_N_4_) membranes supported by a Si frame (Fig. 2 ▸). The Au layer thickness of 120 nm was optimized for high efficiency at around 150 eV (Gd and Tb *N*_4,5_-edges). Each SZP has a diameter of 2 mm, with an outermost zone width of ∼600 nm, and an expected minimum beam waist at 150 eV of the order of 1 µm. A duty cycle of ∼2/3 was selected to improve the first order over 0th order efficiency, with nominal values over the 130–180 eV range of 10 to 16% for the former and 1 to 15% for the latter (Henke *et al.*, 1993 ▸).

The parameters common to all SZPs (*i.e.* apart from their ***s*** value) are summarized in Table 1 ▸.

One of a set of circular apertures (diameter 10, 20 or 50 µm) was inserted at the intermediate focal point of the beamline to generate a secondary source with large transverse coherence, and the bendable KB mirrors were adjusted to have a collimated beam configuration. A fixed 2.5 mm aperture placed before the SZPs ensures a good match of the beam and SZP sizes. Higher diffraction orders are spatially filtered by an OSA placed before the sample at an adjustable distance (1–10 mm). OSAs of different sizes, from 20 µm to 1 mm, are available on the same support and can be interchanged *in situ*. The beam transmitted through the SZP (zero order) and the OSA can be blocked using the movable beamstop in front of the CCD. Test samples are mounted on the XYZR_X_ stage (Fig. 1 ▸), and measurements are performed using the in-vacuum CCD. The spot size produced by the SZP with ***s*** = +1 at the sample position was estimated by knife-edge XZ scans as a function of the SZP coordinate along Y. After optimizing the KB mirror parameters to minimize astigmatism, the smallest spot at 150 eV photon energy was always approximately 3 µm FWHM, a value larger than expected, probably due to residual imperfections in the beam wavefront.

**Figure 3:**
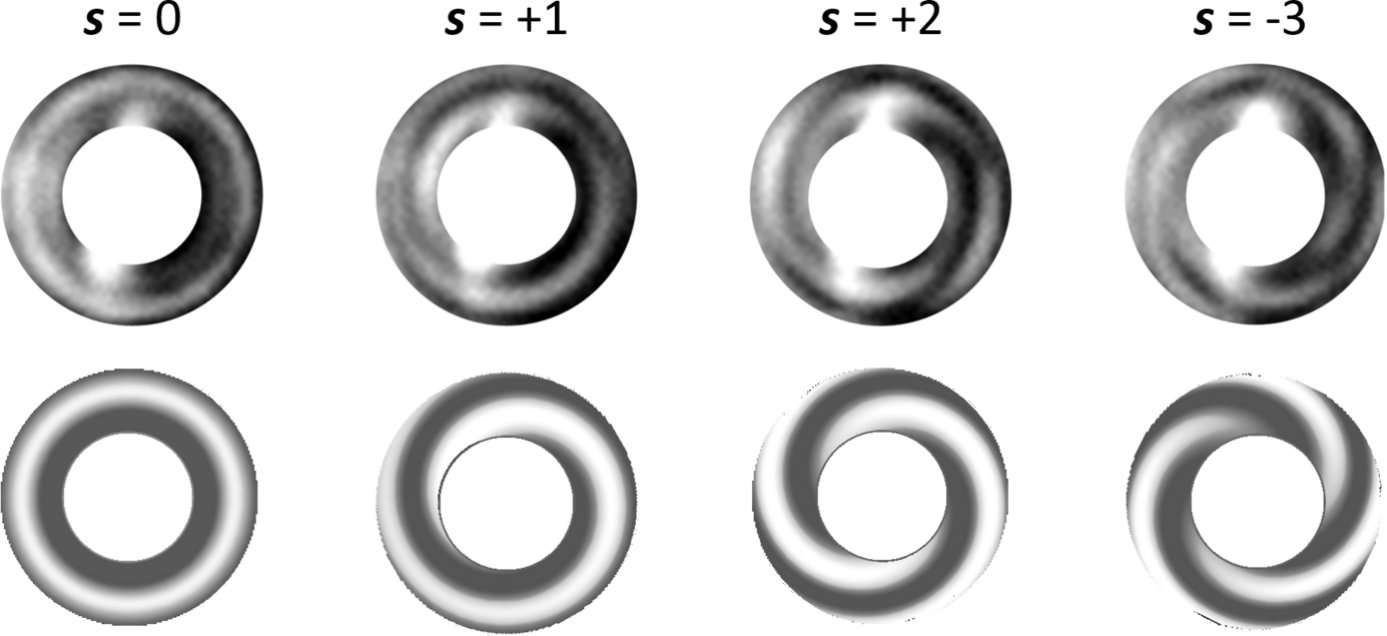
Comparison of experimental (top) and calculated (bottom) intensity modulations arising from the interference between the wave diffracted by an SZP with ***s*** branches (***s*** = 0, 1, 2, −3), having **L** = ***s***, and the wave transmitted through the same SZP, having always **L** = 0.

To test the azimuthal dependence of the phase generated by the SZP, we made use of the interference between the diffracted beam, carrying the desired **L** value, and the beam transmitted through the SZP, which retains the initial **L** = 0 value. Fig. 3 ▸ shows the calculated (bottom) and measured (top) interference patterns for different SZPs (***s*** = 0, +1, +2 and −3). The ***s*** = 0 zone plate produces a ring-shaped intensity pattern due to the variation of optical path between transmitted and diffracted waves. For ***s*** = +1, the additional azimuthal phase modulation leads to a CW spiraling intensity, which becomes a CW two-spirals for ***s*** = +2 and a CCW three-spirals for ***s*** = −3.

**Figure 4:**
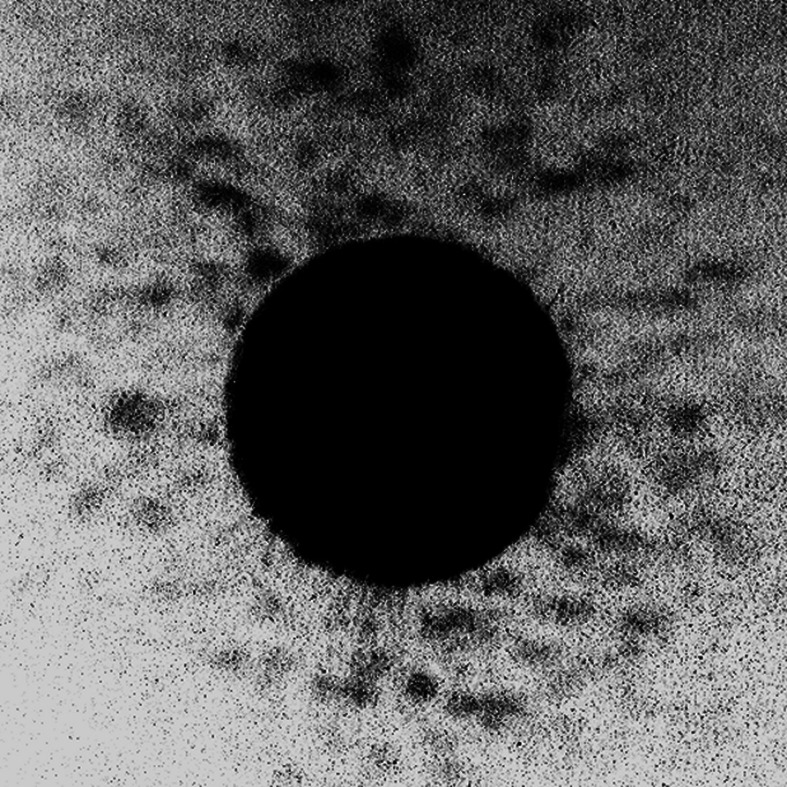
Intensity transmitted through a 50 nm-thick Co_91_Tb_9_ alloy sample using an SZP with ***s*** = +1 at 155 eV (Tb *N*_4,5_-edge). The image (512 pixels, or ∼6.9 mm, wide) was collected with the CCD detector ∼200 mm behind the sample. The intense central spot corresponds to the diverging beam diffracted by the SZP and transmitted through the sample. Speckles observed at larger angles are due to the resonant coherent scattering from the meandric magnetic domain structure.

Fig. 4 ▸ shows the intensity of the 155 eV radiation with **L** = +1 transmitted through a 50 nm-thick Co_91_Tb_9_ alloy sample and collected by the CCD at its closest position, *i.e.* at a sample–CCD distance of ∼200 mm. The central high-intensity disk (saturated scale) corresponds to the diverging beam from the SZP. Its diameter (∼2.8 mm) is consistent with the 2 mm size of the SZP positioned 145 mm upstream of the sample. The weaker intensity modulations (speckle) observed at larger angles are due to resonant scattering at the Tb *N*_4,5_-edge from the up/down magnetization in the meander domains of the perpendicularly magnetized Co_91_Tb_9_ sample.

The SZPs were also used for test experiments in reflectivity mode at the Gd *N*_4,5_-edge. An example is reported in Appendix *A*[App appa].

## 4. Fork gratings

**Figure 5:**
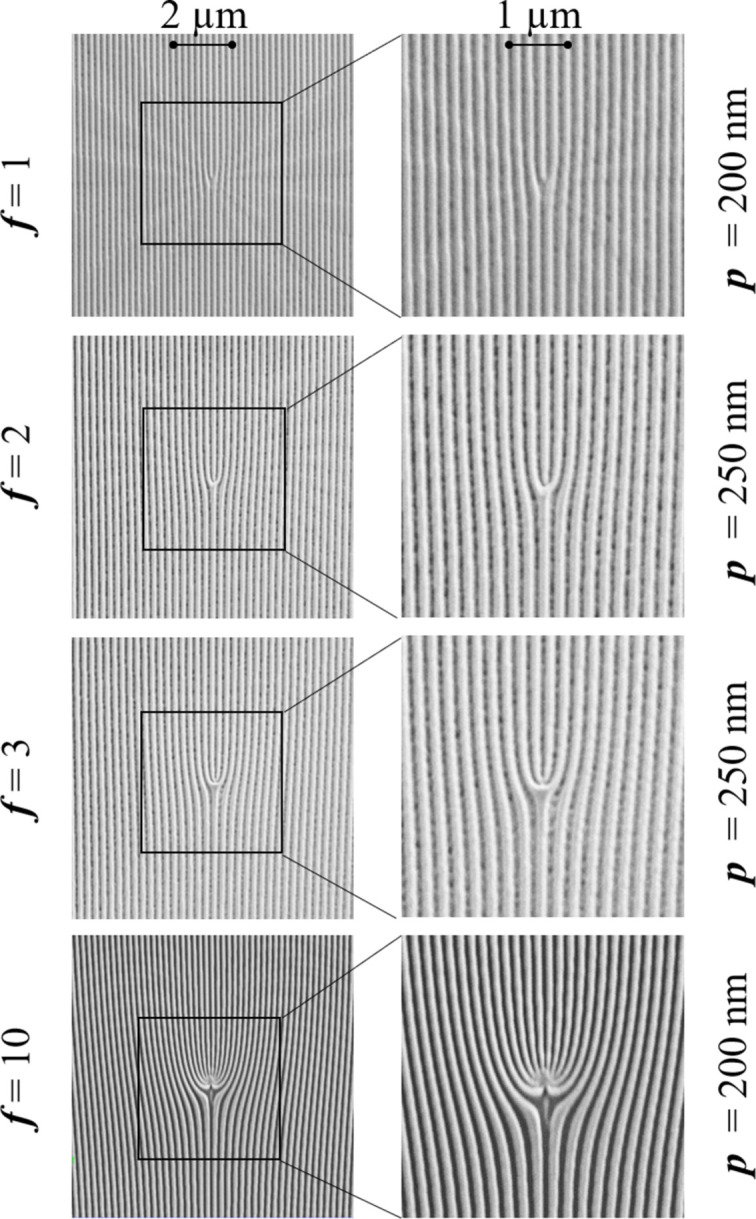
Left: SEM images of four FGs with different ***f*** and ***p*** values. Right: blowups of the central part of the FGs.

FGs (see Fig. 5 ▸) are diffractive elements frequently employed in the visible range to produce OAM beams (Janicijevic & Topuzoski, 2008 ▸; Hu *et al.*, 2022 ▸), and extensions to the X-ray range have already been proposed (Lee *et al.*, 2019 ▸). The OAM value **L** of the produced beam is given by the product of the number ***f*** of additional lines contained in the fork (also referred to as the topological charge of the FG) and the diffraction order ***n***, *i.e.***L** = ***f*** × ***n***. Note that ***f*** can be positive or negative, corresponding to the fork pointing up or down, so that the sign of **L** is determined by the sign of both ***f*** and ***n***. In the following, we will consider only positive ***f*** values.

To test their potential as an alternative to SZPs in the soft X-ray range, we first used a set of small (10 µm × 10 µm) FGs prepared by focused ion beam (FIB) etching of a continuous Au layer deposited on a 100 nm-thick silicon nitride membrane. The grating period ***p*** is either 200, 250 or 600 nm, and the parameter ***f*** varies between 1 and 10, see Fig. 5 ▸. The thickness of the Au layer is 140 nm, optimized for efficient ***n*** = ±1 diffraction over the 140–180 eV energy range, with an angular separation of ∼0.7° between zeroth and first orders for ***p*** = 600 nm.

After optimizing the parameters of the bendable KB mirrors, the beam size was measured to be 40 µm × 30 µm. As a consequence, even when placing the FG at the KB focal position, the beam is larger than the FG active area, and the diffraction orders are affected by scattering from its edges. Although this configuration is not ideal for experiments, it does not affect the conclusions of this test work. Diffraction due to the finite dimension of the fork grating area can be minimized by placing a small pinhole close to the FG, thereby defining a smaller illumination area. The possibility of fabricating larger FGs by lithography and ion-beam etching has already been addressed (see Appendix *B*[App appb]).

**Figure 6:**
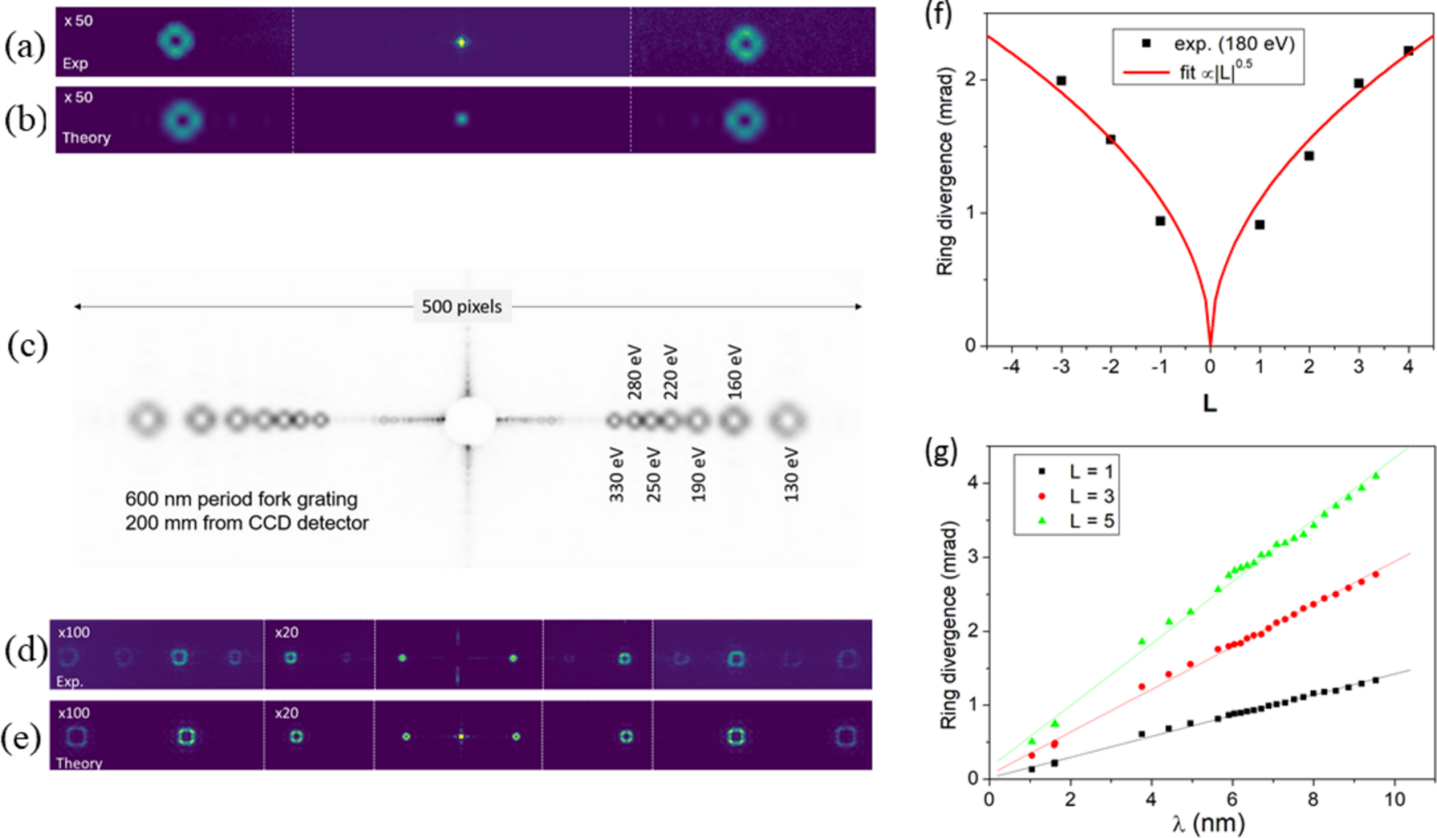
All reported results refer to an FG with ***f*** = 1 and ***p*** = 600 nm. (*a*) Intensity transmitted (***n*** = 0, central spot) and diffracted (***n*** = ±1, multiplied by 50) by the FG at a photon energy of 150 eV. (*b*) Numerical simulation of the results shown in (*a*), using the nominal FG parameters. (*c*) Accumulation of seven scattering patterns (***n*** = ±1) at varying photon energies over the 130–330 eV range, while keeping the positions of the FG and CCD detector fixed. (*d*) Diffraction orders up to ***n*** = ±7 at a photon energy of 778 eV. The ***n*** = 0 beam is blocked by a beam-stop. Relative to ***n*** = 1, the intensity is multiplied by 20 for ***n*** = 2, 3 and by 100 for ***n*** ≥ 4. (*e*) Numerical simulation of the results shown in panel (*d*). (*f*) Points: experimental **θ** values for several diffraction orders at λ ≃ 6.9 nm (180 eV). Line: fit with |**L**|^1/2^ dependence. (*g*) Experimental **θ** values *versus* λ for three diffraction orders over the ∼1–10 nm range, corresponding to the 130–1180 eV photon energy range.

Fig. 6 ▸(*a*) shows that the intensity of the ***n*** = ±1 diffraction peaks exhibits the ring shape expected for a beam with **L** ≠ 0. Numerical simulations of the beam propagation [Fig. 6 ▸(*b*)], performed using the open-source Python package *diffractio* (Sanchez Brea, 2019 ▸), show good agreement with the experimental results in terms of angular position, shape and intensity of the diffracted orders. The intensity scale in Figs. 6 ▸(*a*) and 6(*b*) is multiplied by 50 for the diffracted orders ***n*** = ±1. The integrated intensity of the ***n*** = ±1 orders is about 9% of that measured for ***n*** = 0, a ratio lower than the calculated 14.5% from the data in Fig. 6 ▸(*b*). This discrepancy is due to several factors: the incoming beam is larger than the FG, the surrounding Au layer is partially transparent at a photon energy of 150 eV, imperfections in the FG bar shape and duty cycle, and the possible presence of higher-order contamination from the X-ray beamline. All these contributions affect only the ***n*** = 0 intensity.

Unlike SZPs, FGs do not act as focusing elements. Combined with the use of a 2D detector, this makes it easier to vary the photon energy without moving other mechanical components (a lateral translation of the sample may still be required, depending on its size and homogeneity). Fig. 6 ▸(*c*) shows the ***n*** = ±1 diffracted intensity collected by the CCD detector, kept at a fixed position, for seven different photon energies in the 130–330 eV range. By adjusting the CCD distance and accepting a lower efficiency of the FG, diffraction patterns were also collected at photon energies well above the design range, up to approximately 1200 eV. Fig. 6 ▸(*d*) shows an example measured at 778 eV, corresponding to the Co *L*_3_ edge associated with a 2*p*-to-3*d* electron excitation (note that the intensity of the ***n*** ≠ 1 diffracted beams has been multiplied by either 20 or 100). Numerical simulations again reproduce the experimental results well [Fig. 6 ▸(*e*)]. In particular, the distortion of the ring shape due to scattering from the edges of the small grating area, especially pronounced at high diffraction orders, is correctly reproduced by considering in the simulations a square FG (10 µm) smaller than the incoming beam size (40 µm). The absence in the simulations of the faint even diffraction orders observed in the measurements is due to the assumption of perfectly rectangular bars with exactly half-period width, a condition not strictly fulfilled by the real grating.

To each diffracted beam with OAM value **L** = ***f*** × ***n*** is associated a divergence **θ**, defined as the angle subtended by the ring of maximum intensity. This divergence is expected to be proportional to λ|**L**|^1/2^, where λ is the X-ray wavelength (note that deviations from the 0.5 exponent have been reported in the optical range; Karimi *et al.*, 2007 ▸). Fig. 6 ▸(*f*) compares the measured divergence for several diffraction orders as a function of their expected **L** value with the square root dependence (red line). Fig. 6 ▸(*g*) shows the measured **θ**(λ) values for three diffraction orders, exhibiting a nearly linear dependence over the 130–1180 eV photon energy range. Together with the FG-to-sample distance, the divergence **θ** defines the size of the OAM beam at the sample position. Since **θ** depends on ***f***, ***n*** and λ, the beam size can vary over a wide range. According to Fig. 6 ▸(*g*), for a given FG with ***p*** = 600 nm and ***f*** = 1 placed 120 mm upstream of the sample, extreme values range from ∼20 µm (1200 eV, ***n*** = 1, **θ** ≃ 135 µrad) to ∼0.5 mm (130 eV, ***n*** = 5, **θ** ≃ 4 mrad). The separation between diffraction orders is ∼12 mrad, which is sufficient to select a single order by using the OSA. To control the beam size at the sample position, one can also adjust the FG-to-sample distance. In our tests this parameter was constrained by the focal distance of the SZPs, since both devices shared the same holder; however, in principle, it can be reduced to values as low as ∼30 mm.

**Figure 7:**
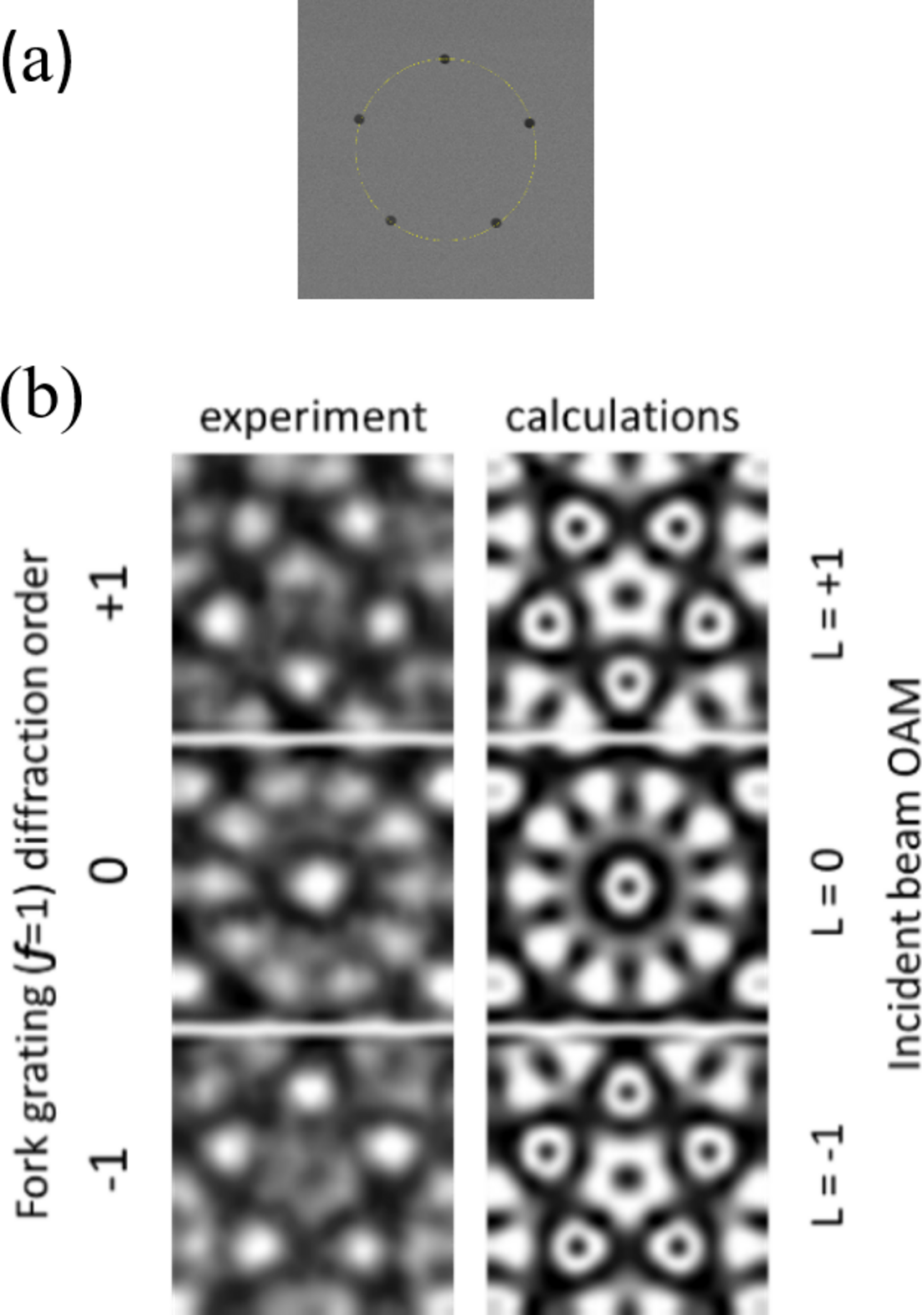
(*a*) SEM image of the opaque mask with five apertures at the vertices of a pentagon inscribed in a circle with a diameter of 20 µm. (*b*) Left column: experimental interference patterns obtained at λ = 1.59 nm (778 eV) using the ***n*** = −1, 0, +1 diffraction orders of the FG (***f*** = 1, ***p*** = 600 nm). Right column: calculated patterns for an incoming beam with **L** = −1, 0, +1.

In addition to confirming the expected radial distribution of the intensity [Figs. 6 ▸(*a*) and 6(*d*)], we also verified the azimuthal phase dependence for an **L** ≠ 0 beam diffracted by an FG by measuring the interference pattern generated by scattering through an opaque mask with five apertures (∼1 µm in diameter) placed at the vertices of a pentagon [Fig. 7 ▸(*a*)], using an approach similar to that reported by Hickmann *et al.* (2011 ▸).

In Fig. 7 ▸(*b*), the interference patterns measured by illuminating the mask with diffraction orders ***n*** = −1, 0, +1 of the FG (***f*** = 1) are compared with the far-field interference patterns calculated for incident beams with **L** = −1, 0, +1. The good agreement between experiments (*versus****n***) and calculations (*versus***L**) confirms that the beams generated by the FG possess the expected azimuthal dependence of the phase.

## 5. OAM arithmetic with SZP and FG devices

Here we examine the case of an FG being illuminated by a beam that already carries an OAM (**L** ≠ 0), generated either by an SZP or by another FG, and show that one can establish an *integer arithmetic* for soft X-ray OAM beams. A similar approach has already been proposed for optical laser radiation (Liu *et al.*, 2025 ▸); we also note that a very recent article (Khan *et al.*, 2026 ▸) reports the results of a similar detailed study, including supporting model calculations.

**Figure 8:**
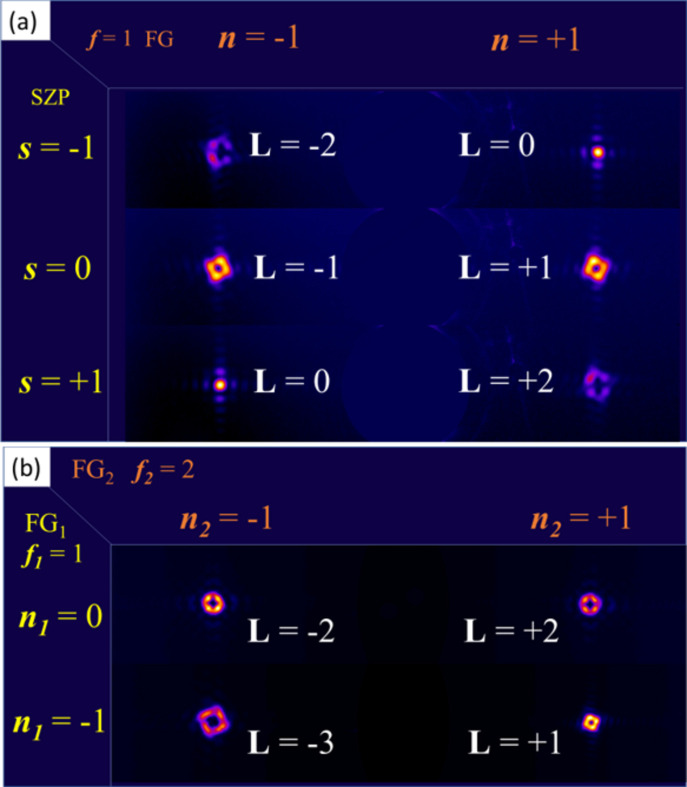
(*a*) Diffraction orders ***n*** = ±1 generated by the FG (***f*** = 1), illuminated using an SZP with ***s*** = −1 (top), ***s*** = 0 (middle) and ***s*** = +1 (bottom). (*b*) Intensity pattern obtained by aligning two fork gratings, FG_1_ and FG_2_, in cascade: the first with ***f***_1_ = 1 and the second with ***f***_2_ = 2. FG_2_ is illuminated either by the zeroth order of FG_1_ (***n***_1_ = 0, top) or by its first negative order (***n***_1_ = −1, bottom). The expected **L** values for each spot at the detector are indicated in white. The photon energy is 152 eV in all cases.

Fig. 8 ▸(*a*) illustrates the case in which the incoming radiation (152 eV, λ = 8.15 nm), produced by an SZP and carrying an OAM value **L** = ***s*** of 0, +1 or −1, impinges on an FG with ***f*** = 1, generating diffraction peaks of order ***n*** = ±1. For an incoming **L** = 0 beam (middle image), the two rings corresponding to ***n*** = ±1 diffraction from the FG have the same size, associated with the expected **L** = ±1 values (see Fig. 7 ▸). Using an SZP with ***s*** = +1, imparting a value **L** = +1 to the incoming beam (bottom image), the ***n*** = +1 diffraction generates a wider ring [*i.e.* a larger **L** value according to Fig. 6 ▸(*f*)], while ***n*** = −1 produces a closed spot instead of a ring, which we associate with an **L** = 0 beam. The opposite behavior is observed when using an SZP generating an incoming **L** = −1 beam (top image). These results show that the FG adds a value Δ**L** = ***n*** × ***f*** to the incoming beam, regardless of its initial **L** value.

The same approach can make use of two FGs used in sequence, as shown in Fig. 8 ▸(*b*). Here, a first grating, FG_1_, with ***f***_1_ = 1 and ***p***_1_ = 600 nm, is followed by a second grating, FG_2_, with ***f***_2_ = 2 and ***p***_2_ = 250 nm. When FG_2_ is illuminated by the zeroth order of FG_1_ (***n***_1_ = 0), its ***n***_2_ = ±1 diffraction orders produce two equivalent rings with expected **L** = ±2 values [Fig. 8 ▸(*b*), top]. Illuminating FG_2_ with the ***n***_1_ = −1 beam (**L** = −1) results in a wider ring for ***n***_2_ = −1 and a narrower ring for ***n***_2_ = +1 [Fig. 8 ▸(*b*), bottom], which we interpret as the result of the sum of the OAM values imparted by each diffraction event, finally leading to a beam with **L** = ***n***_1_× ***f***_1_ + ***n***_2_ × ***f***_2_.

Taken together, these two examples (*i.e.* SZP+FG and FG+FG) demonstrate that combining several devices (either SZPs or FGs) in cascade introduces an integer OAM arithmetic, as previously proposed in the visible range (Ruffato *et al.*, 2019 ▸; Liu *et al.*, 2025 ▸). At the cost of introducing an additional diffractive element, *i.e.* at the expense of photon flux, OAM arithmetic adds flexibility in controlling the final **L** value of the resulting X-ray beam when starting from a limited number of FG or SZP devices.

It is also worth noting that the set of results reported in Fig. 8 ▸ shows that a single FG device can be used for a simple, efficient and reliable determination of the **L** value of both the incoming and scattered X-ray beams. We will make use of this capability in the next section.

## 6. Harmonic emission from a helical undulator

Several years ago (Sasaki *et al.*, 2007 ▸; Sasaki & McNulty, 2008 ▸), it was predicted that the circularly polarized light produced by helical undulators (HUs) carries, in addition to the spin angular momentum of value **σ** (in ℏ units) defined by the sign of the helicity (**σ** = ±1), an OAM of value **L** = **σ**(**h** − 1), where **h** ≥ 1 is the harmonic order of the undulator emission (**h** = 1 for the fundamental). This was experimentally demonstrated at both synchrotron (Sasaki *et al.*, 2007 ▸; Bahrdt *et al.*, 2013 ▸) and FEL (Hemsing *et al.*, 2014 ▸; Rebernik Ribič *et al.*, 2017 ▸) sources. The characterization of the OAM value of the HU emission has mostly been performed under out-of-focus conditions and with minimal intervention of optical elements. Here, we investigate this property at the experimental chamber located at the end of the beamline.

The SEXTANTS beamline is equipped with two HUs of different periods to cover the full energy range in fundamental emission; however, for radiation security reasons, the two devices cannot be operated simultaneously. Therefore, the standard test of detecting the OAM value, based on the interference of same-wavelength emission at, for example, **h** = 1 and **h** = 2 from distinct devices (Bahrdt *et al.*, 2013 ▸), cannot be performed at SEXTANTS. Using the helical configuration of a single undulator (*i.e.* |**σ**| = 1), we carried out three alternative tests, at 155 eV and at 780 eV, based on the results shown in Sections 4 and 5:

(*a*) Comparison, at the same photon energy and in circular polarization mode, of the intensity distributions for **h** = 1 and **h** = 2, expected to produce Gaussian and ring-shaped beams, respectively. These tests provided no clear evidence of ring-shaped intensity at or near the focal plane for **h** = 2.

(*b*) Comparison of the intensity distributions for **h** = 1 and **h** = 2 after scattering from the five-aperture mask shown in Fig. 7 ▸(*a*). In our measurements, we did not observe any change in the interference pattern comparable with that shown in Fig. 7 ▸(*b*).

(*c*) Comparison of the intensity patterns generated by an FG (***f*** = 1, ***n*** = ±1) for **h** = 1 (expected **L** = 0) and **h** = 2 (expected **L** = 1) incoming circularly polarized beams. In this case as well, we did not observe any variation similar to those reported in Fig. 8 ▸(*b*); *i.e.* we found no clear indication of an incoming beam with **L** ≠ 0.

Summarizing, all checks performed so far at the level of the experimental chamber failed to demonstrate a well defined **L** value for the **h** = 2 circularly polarized HU emission. As mentioned above, previous experiments demonstrating the OAM value in the HU emission were performed out of focus and with little or no intervention of optical elements. It is therefore likely that the well defined **L** value of the diverging beam at the source is degraded during radiation transport and focusing along the beamline, where, at SEXTANTS, it encounters two slits, six mirrors and a grating before reaching its final destination at the IRMA-2 experimental chamber.

We conclude that, at present, we are not in a position to propose the use of radiation from the SEXTANTS HU sources for OAM-dependent experiments. Further tests will be carried out by the beamline staff, in collaboration with the Source and Optics groups at Synchrotron SOLEIL, to determine whether the beam transport and focusing conditions can be controlled and adapted to preserve the OAM of the X-rays down to the experimental stations.

## 7. Fourier transform holography with X-OAM beams

As a first pilot experiment with OAM X-ray beams at SEXTANTS, we performed imaging of magnetic materials via resonant scattering of coherent X-rays, using the Fourier transform holography (FTH; Eisebitt *et al.*, 2004 ▸) method already implemented at the beamline (Sacchi *et al.*, 2012 ▸; Popescu *et al.*, 2019 ▸). This allows us to better define the conditions for future user experiments by assessing the feasibility of element-selective magnetic imaging with OAM-carrying X-rays in terms of setup features, beam characteristics and the ability to span a wide energy range.

We investigated two samples: the 50 nm-thick Co_91_Tb_9_ alloy film already discussed in Section 3, and a (Co_1nm_/Gd_0.5nm_) × 40 multilayer deposited on a 100 nm-thick Si_3_N_4_ membrane and patterned by FIB etching into a regular array of ∼400 nm × 400 nm squares separated by ∼100 nm wide lines. The CoGd sample also featured a 1 µm-thick Au holography mask integrated on the opposite side of the Si_3_N_4_ membrane.

**Figure 9:**
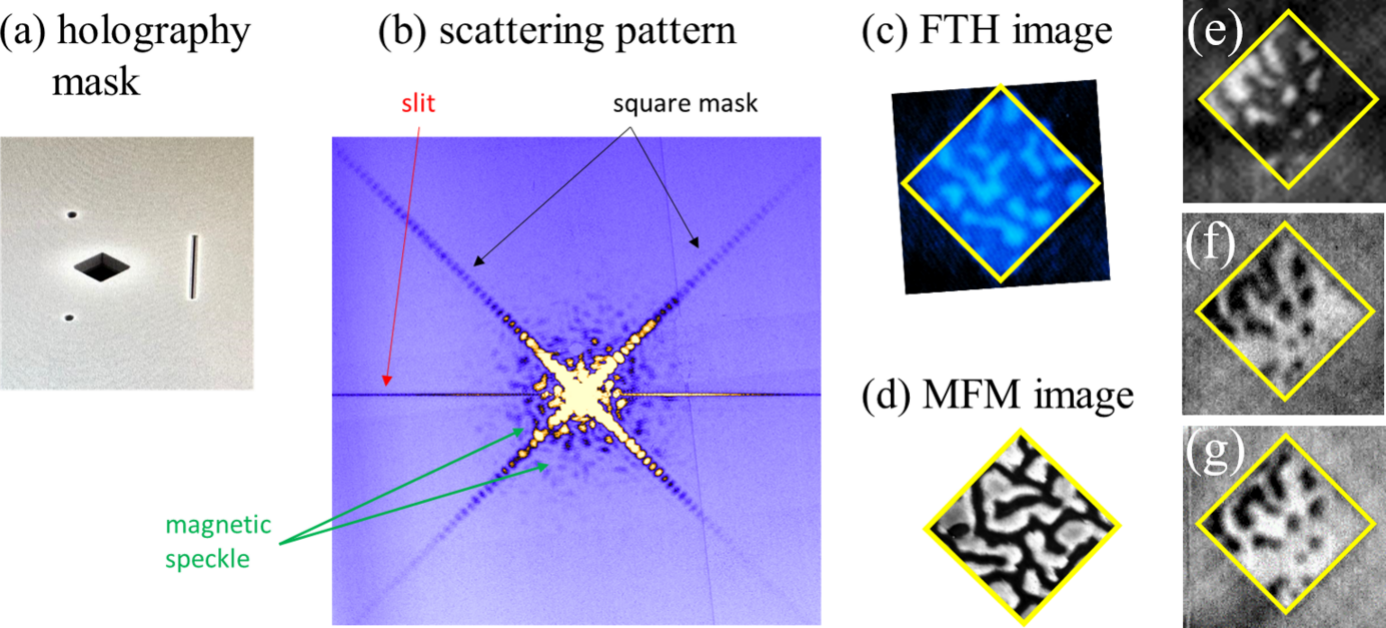
(*a*) Holography mask (1 µm Au) with a 2.4 µm square aperture, two point-references, and a slit, placed on top of the 50 nm thick Co_91_Tb_9_ sample. (*b*) Scattering pattern collected at 778 eV (Co *L*_3_ resonance) using a photon beam with right circular polarization and **L** = +1. Arrows associate different scattering features with sample and mask characteristics. (*c*) Image of the perpendicular magnetic domains obtained by taking the difference between left- and right-circular polarization measurements. (*d*) MFM image of the same sample acquired in a different region of the surface. (*e*–*g*) FTH images of magnetic domains in the Co_91_Tb_9_ sample obtained at the Tb 4*d*–4*f* resonance [(*e*), **L** = −1] and at the Co 2*p*–3*d* resonance [(*f*), **L** = −1, and (*g*), **L** = +1]. Diamonds indicate the size of the object aperture. The imaged sample area differs between (*c*) and (*e*–*g*).

As shown in Section 4 and Fig. 6 ▸, FGs optimized for operation at the Gd and Tb 4*d* resonances also provide sufficient intensity at the Co 2*p* resonance. A holographic mask [Fig. 9 ▸(*a*)] was placed on top of the CoTb sample, and scattering patterns such as the one shown in Fig. 9 ▸(*b*) were collected as a function of photon energy (155 and 778 eV), light polarization (left/right circular) and **L** values (0, ±1), using the same FG with ***f*** = 1 and ***p*** = 600 nm. The magnetic image in Fig. 9 ▸(*c*) was obtained at the Co 2*p* resonance for **L** = +1, by taking the Fourier transform of the difference between scattering patterns acquired with opposite helicities. It is compared with an MFM image of the same size [Fig. 9 ▸(*d*)], previously obtained from a different region of the same sample, showing a similar magnetic domain structure but different details. Results obtained for **L** = ±1 at the Tb 4*d* (153 eV) and Co 2*p* (778 eV) resonances are summarized and compared in Figs. 9 ▸(*e*)–9(*g*).

**Figure 10:**
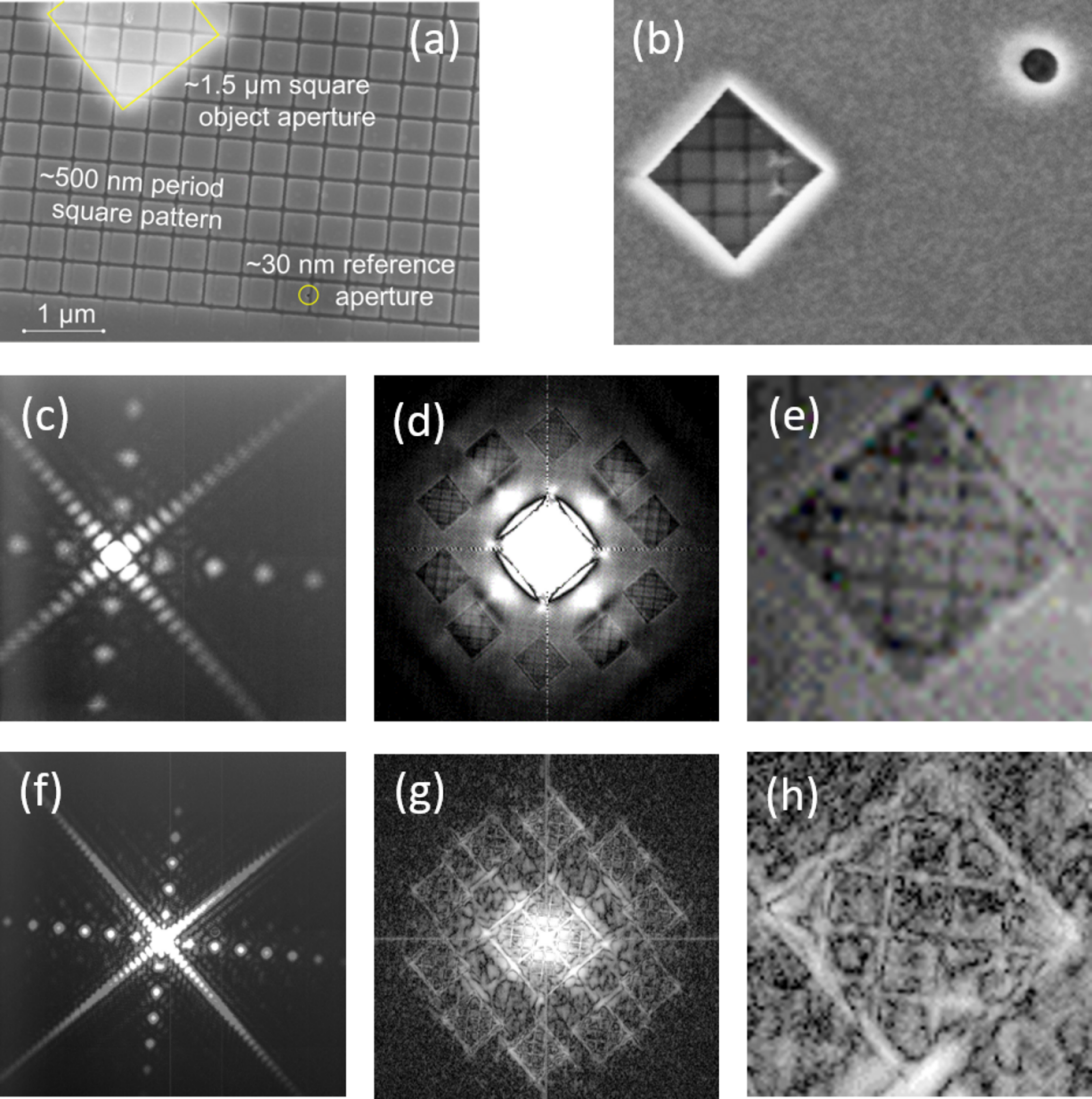
(*a*, *b*) SEM images of the CoGd test sample with an integrated 1 µm-thick Au holography mask. Image (*a*), showing the patterned CoGd side, also displays the object aperture (lighter area, top left) and one reference aperture (circled, lower right), both outlined in yellow. Similarly, image (*b*), showing the Au mask side, displays the object aperture, with the pattern visible in transmission, and one of the point references (starting with a larger diameter on the Au side). (*c*, *f*) Scattered intensity from the patterned CoGd sample measured at 150 eV [(*c*), Gd *N*_4,5_ resonance] and at 1185 eV [(*f*), Gd *M*_5_ resonance], using **L** = +1 radiation (FG with ***p*** = 600 nm and ***f*** = 1). (*d*, *g*) Corresponding FTH images. (*e*, *h*) Detail of an image of the object (1.5 µm × 1.5 µm) produced by one of the reference apertures. The square pattern inside the object has a period of ∼500 nm.

We also tested the possibility of accessing the rare-earth 3*d* resonances in the 1170–1250 eV energy range. Fig. 10 ▸ shows results of FTH imaging obtained for the CoGd sample. SEM images display the square pattern on the CoGd multilayer side [Fig. 10 ▸(*a*)] and the object and reference apertures on the Au mask side[Fig. 10 ▸(*b*)]. Both *N*_4,5_ and *M*_5_ Gd resonances were measured using the same FG optimized for 150 eV, changing only the undulator source and the CCD distance from the sample. The quality and intensity of the scattering signal, Figs. 10 ▸(*a*) and 10(*d*), are sufficiently good at both resonances; however, since this sample was not designed for operation at such high photon energies, the 1 µm thickness of the Au mask is not sufficient to suppress transmission at ∼1200 eV. As a consequence, the FTH-reconstructed images obtained at the Gd *M*_5_ resonance are of significantly lower quality.

Overall, these results are very promising for performing **L**-dependent magnetic imaging experiments using holography and ptychography (Pancaldi *et al.*, 2024 ▸). The latter technique is more time-consuming because it requires raster scanning of the sample, but it is less demanding in terms of photon flux per image than holography, which relies on the intensity transmitted through very small reference apertures.

Testing the use of X-OAM beams for FTH imaging is of interest for two main reasons, related to the radial intensity distribution and azimuthal phase dependence of these beams. The former allows for a more favorable distribution of intensity over the object and reference apertures, whose area difference can easily exceed four orders of magnitude. Since FTH is based on the interference between beams scattered by the references and by the object, placing the references on the ring of maximum intensity and the object on the beam axis results in a better balance of their relative scattered amplitudes, which is beneficial for achieving high-visibility interference patterns and, ultimately, higher-quality images. The role of the azimuthal phase dependence in FTH is more difficult to assess: calculations predict an influence on the interference pattern, but this has not yet been verified experimentally, and further work is required to exploit this property of X-OAM beams.

## 8. Conclusions

We have developed and implemented a new experimental setup within the IRMA-2 scattering chamber of the SEXTANTS beamline at Synchrotron SOLEIL. This setup enables transmission and reflection measurements using soft X-ray radiation with OAM, generated either by SZPs or by FGs, depending on the specific experimental requirements. Both SZPs and FGs are chromatic diffractive devices that, in principle, require adjustment of both sample and detector positions when varying the photon energy. SZPs also act as focusing elements and should be preferred when a small (sub-µm) spot size is required. FGs, on the other hand, facilitate the use of higher diffraction orders, enabling experiments over a broader range of OAM values. Moreover, scanning of the sample and detector is not necessarily required when using FGs, provided that the experiment is conducted on sufficiently large and homogeneous samples and with large 2D detectors. From our test experiments, the most important characteristic distinguishing FGs from SZPs is that they are not focusing devices. This allowed us to perform measurements using a single FG while spanning one decade in photon energy (covering the Gd 4*d*, Co 2*p* and Gd 3*d* resonances) without breaking vacuum, simply by adjusting the *in situ* positions of the sample and detector. This would not have been possible with the SZPs used here, since their focal distance changes significantly with photon energy (from 14 cm at the Gd 4*d* resonance to 75 cm at the Co 2*p* and 1.15 m at the Gd 3*d* resonances). The ability to cover a wide energy range using a single device and a single experimental configuration, without the need to access the vacuum vessel to replace or adjust optical elements, is particularly attractive for spectroscopy experiments at synchrotron facilities.

In terms of photon flux, we observe an overall loss of about two orders of magnitude, from ∼10^13^ to ∼10^11^ photons s^−1^, for both SZPs and FGs. This estimate is consistent with expected losses due to device efficiency (10–15% in first-order diffraction for both SZPs and FGs), combined with an additional loss of about one order of magnitude due to the creation of a secondary source in the case of SZPs and to the mismatch between the incoming beam size and the device dimensions in the case of FGs.

We have performed test experiments based on resonant magnetic scattering (Figs. 4 ▸, 11 ▸ and 12 ▸) and imaging by Fourier transform holography (Figs. 9 ▸ and 10 ▸). These results show that even flux-demanding experiments, such as magnetic imaging by FTH, can be carried out while spanning approximately one decade in photon energy. Our findings demonstrate that, using a single FG and standard beamline components (helical undulators, monochromator and focusing optics), it is possible to generate soft X-ray beams with independently controlled spin and orbital angular momentum over a continuous and wide photon energy range. In particular, the 130–1200 eV range explored here covers most of the relevant resonances of multi-element transition-metal/rare-earth magnetic systems.

The combination of multiple SZP and/or FG devices in sequence provides a means to tailor the final **L** value. Together with the choice of diffraction order for FGs, this introduces additional flexibility in defining the OAM of the resulting X-ray beam.

FGs have also proven to be effective tools for simple and reliable characterization of the OAM content of incoming beams, offering an efficient diagnostic method for synchrotron and free-electron laser X-ray sources.

**Figure 14:**
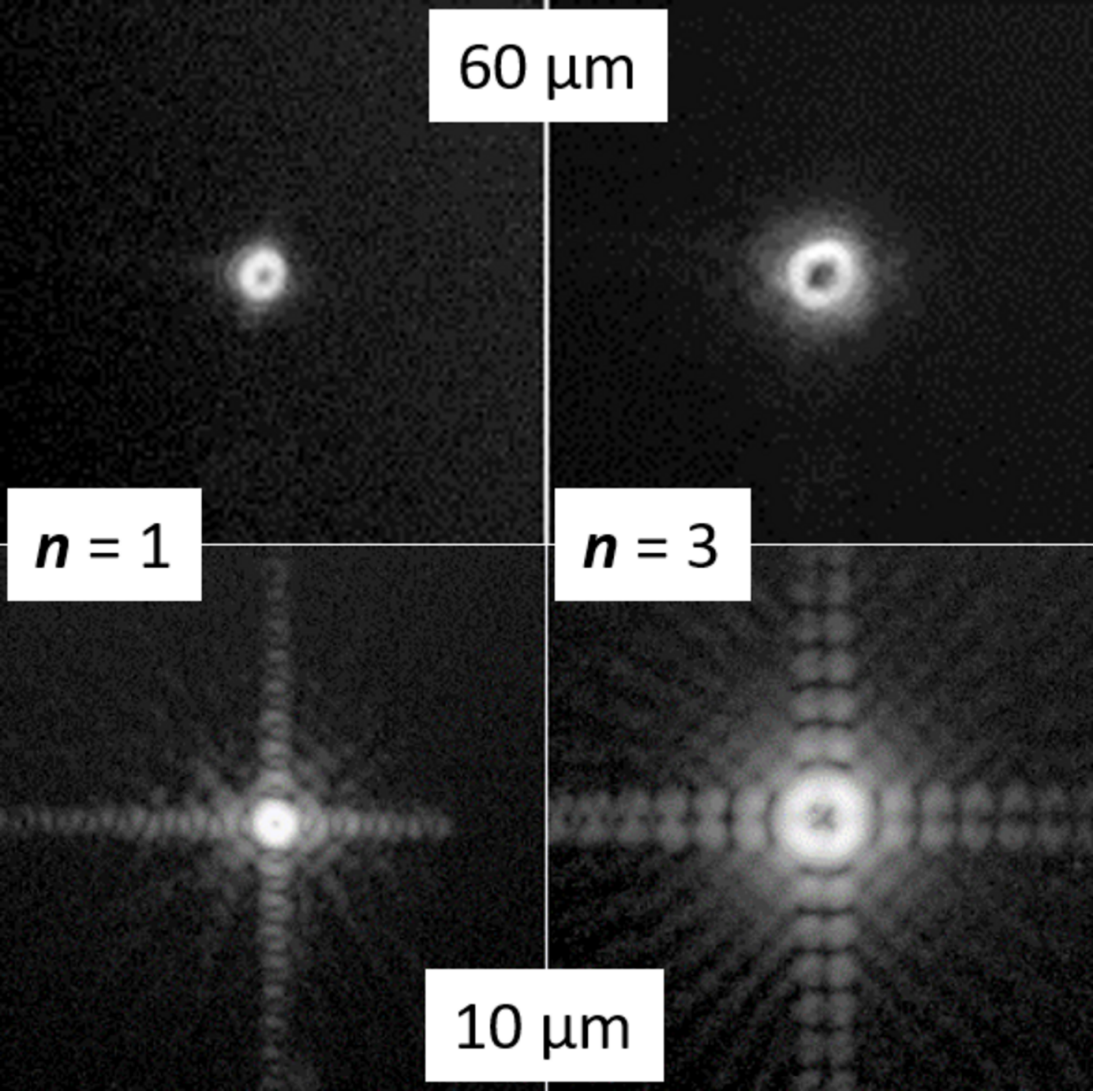
Diffraction from square FGs with ***f*** = 1, ***p*** = 200 nm, and a lateral size of 60 µm (top) or 10 µm (bottom). The intensity of diffraction orders ***n*** = 1 (left) and ***n*** = 3 (right) is shown on a logarithmic scale.

The FGs used in these experiments are prototype devices. In particular, their limited area constrained their performance, which can be significantly improved through further optimization, as illustrated in Fig. 14. We are therefore confident in the technical feasibility of magnetic scattering and coherent imaging experiments with soft X-ray OAM beams at the SEXTANTS beamline.

Finally, it is worth noting that Synchrotron SOLEIL is undergoing a major technical upgrade that will impact, among other parameters, the coherent flux of the source—a key factor for optimizing the delivery of soft X-ray beams with controlled orbital angular momentum.

## Figures and Tables

**Figure 11:**
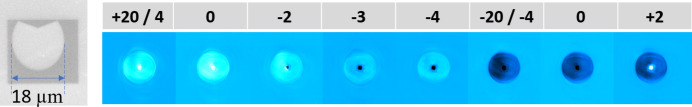
Normalized reflectivity from an 18 µm pacman-shaped Co/Gd dot (SEM image on the left), as a function of the applied magnetic field (in mT, indicated above each image). Measurement conditions: SZP **L** = +1, OSA 0.5 mm, photon energy 152 eV (Gd 4*d* resonance), incidence angle 45°, linear *p*-polarization (vertical). Each image consists of 1024 × 1024 pixels, corresponding to a width of ∼13.8 mm.

**Figure 12:**
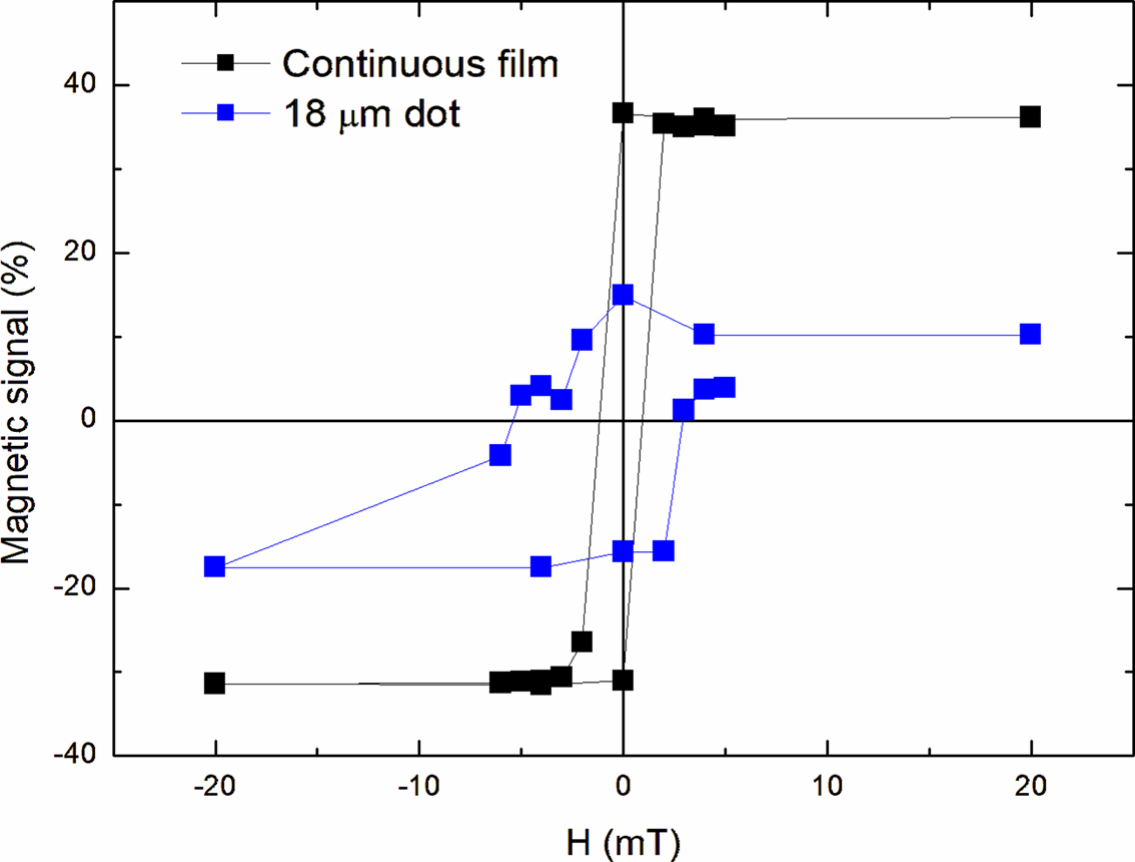
Hysteresis curves obtained selecting the magnetic signal corresponding to the 18 µm dot (large disk area, X-rays with **L** = 1) and to the continuous film (small central spot, X-rays with **L** = 0). The magnetic signal is defined as [*I*(*H*) − *I*_Ave_]/*I*_Ave_, where *I*_Ave_ is the average signal over a complete cycle.

**Figure 13:**
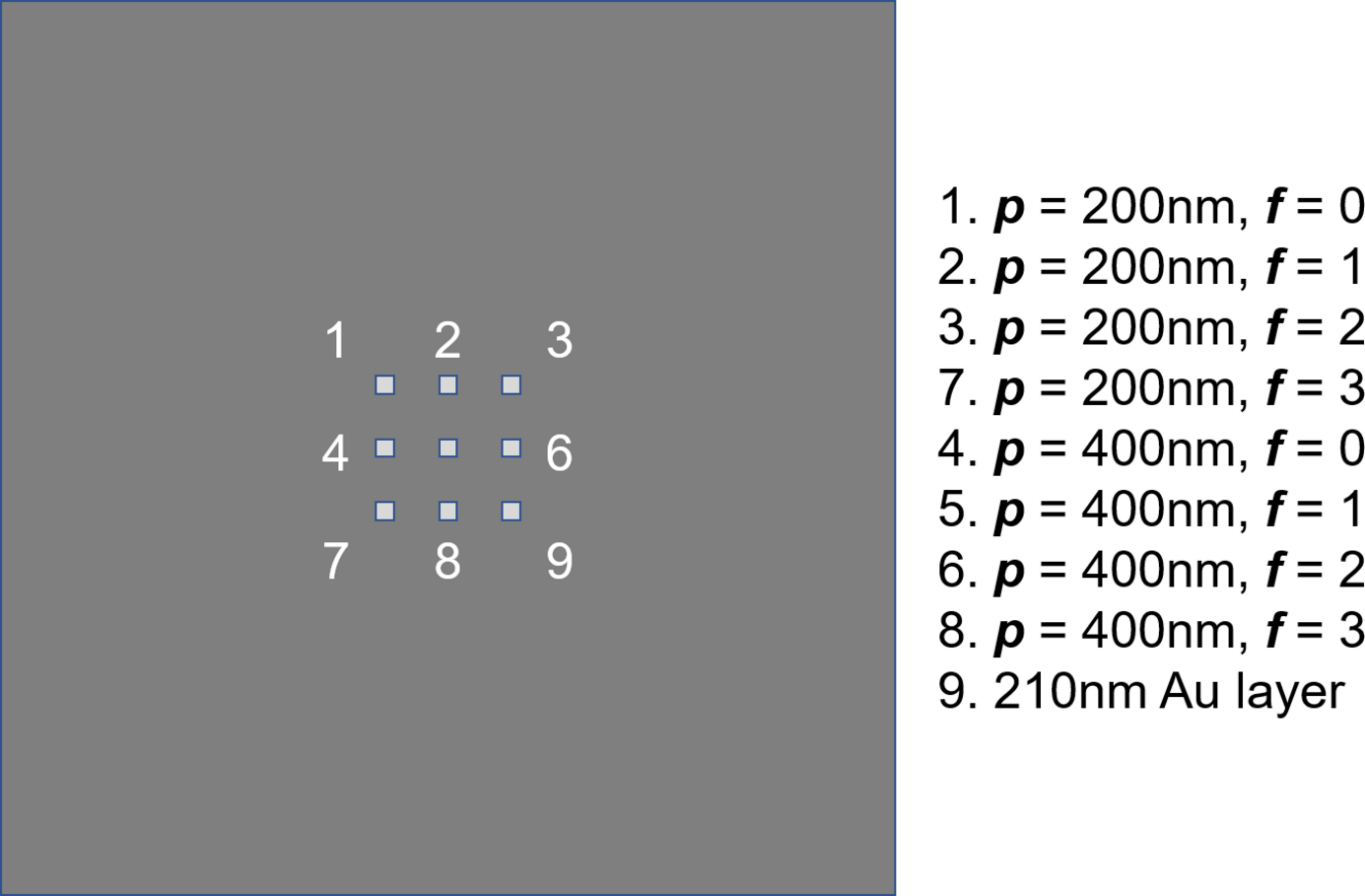
Sketch of a Si chip (7.5 mm wide) supporting nine Si_3_N_4_ square membranes (150 µm wide, 200 nm thick, 530 µm pitch), containing eight Au FGs and one continuous Au layer (210 nm thick). The area of each FG is 60 µm × 60 µm, and their ***p*** and ***f*** values are indicated on the side.

**Table 1:** Design parameters common to all SZPs

Si frame size	10 mm × 10 mm
Si_3_N_4_ membrane size	2.5 mm × 2.5 mm
Si_3_N_4_ membrane thickness	50 nm
Cr coating	5 nm
Au film thickness	120 nm
SZP diameter	2 mm
SZP smaller outer zone	∼600 nm
SZP duty cycle	∼2/3
Focal distance @ 150 eV	∼145 mm
Spot size FWHM	∼1 µm

## Data Availability

Data supporting the results reported in this article are available upon reasonable request to the corresponding authors.

## Appendix A Magnetic reflectivity with an SZP-generated OAM beam

Using the focused beam generated by the ***s*** = +1 SZP, we also performed high-angle reflectivity measurements, with the CCD detector mounted along the vertical axis. The main objective of this test was to assess the feasibility, at SEXTANTS, of using X-rays with controlled polarization and OAM values for flux-demanding measurements, where reflectivities on the order of 10^−6^ are expected (∼150 eV, *p*-polarization, angles close to the Brewster extinction condition). This scattering geometry has also been employed in previous experiments with OAM beams (Fanciulli *et al.*, 2025 ▸) and it is therefore of potential interest for users. The sample was a (Co_1nm_/Gd_0.5nm_) × 20 multilayer deposited on a Si substrate, in which a pacman-shaped 18 µm dot was isolated by FIB etching (Fig. 11 ▸, left). The 152 eV (Gd 4*d* resonance) X-ray beam was *p*-polarized (linear polarization oriented in the vertical scattering plane) and impinged at ∼45° on the sample. The reflected intensity around a 90° scattering angle (CCD images in Fig. 11 ▸) was measured for different values of the applied magnetic field and normalized to the average intensity over a full hysteresis cycle 20 mT wide. The large intensity disk (∼6.3 mm in diameter) observed in each image corresponds to the SZP-diffracted beam with **L** = +1, fully focused onto the Co/Gd dot and specularly reflected toward the CCD positioned 450 mm from the sample. At the center of each image, a smaller spot corresponding to the **L** = 0 beam transmitted through both the SZP and the square 0.5 mm OSA is also observed; its intensity is dominated by interaction with the continuous (*i.e.* unpatterned over the illuminated area) Co/Gd layer surrounding the 18 µm dot. The data show that, as a function of the applied field—particularly between 2 and 4 mT—the magnetization inside the dot behaves differently from that of the surrounding continuous layer. Hysteresis curves obtained by selecting the signal from the dot (large disk) and from the continuous layer (small central spot) are compared in Fig. 12 ▸. Measurements of this type enable experiments on magnetic helicoidal dichroism similar to those already performed at free-electron laser sources, both in static (Fanciulli *et al.*, 2022 ▸) and pump–probe (Fanciulli *et al.*, 2025 ▸) modes.

## Appendix B First tests of larger FGs

A new set of FGs with larger size and optimized for operation over the 600–1300 eV energy range has been fabricated at Institut Néel, Grenoble, France, and briefly tested at the SEXTANTS beamline. Eight FGs with ***p*** = 200 nm or 400 nm and ***f*** = 0, 1, 2, 3 are prepared on separate 200 nm-thick Si_3_N_4_ membranes mounted on a single Si frame, according to the scheme shown in Fig. 13 ▸. Each FG is fabricated by electron-beam evaporation of a 210 nm Au layer onto a 60 µm × 60 µm square at the center of each membrane, followed by electron-beam lithography, Ti mask deposition and lift-off, and finally ion-beam etching.

The intensity measured at the first and third diffraction orders using an X-ray beam ∼40 µm × 30 µm in size is shown in Fig. 14 ▸ (note the logarithmic scale) and compared with analogous measurements obtained using a 10 µm size FG (see Section 4). The strong diffraction from the edges observed for the latter case is absent, or at least strongly reduced, for the larger 60 µm FG.
